# Two key algorithms for intelligent inspection robots in electric bicycle charging sheds

**DOI:** 10.1038/s41598-025-99825-9

**Published:** 2025-05-05

**Authors:** Yingjian An, Ge Wei

**Affiliations:** 1 Shanghai Construction Management Vocational Technical College, No. 588 Gaojing Road, Qingpu District, Shanghai, People’s Republic of China; 2 Shanghai Communications Polytechnic, Shanghai, People’s Republic of China

**Keywords:** Intelligent inspection robot, RRT* algorithm, SOLOv2 algorithm, Charging sheds, Information technology, Software

## Abstract

The deployment of intelligent inspection robots in electric bicycle charging sheds is critical for preventing fire hazards, yet faces challenges in navigating narrow passages and recognizing small components. This paper proposes two enhanced algorithms to address these issues: (1) a multi-root node RRT* (MS-RRT*) for efficient narrow-channel path planning, and (2) an improved SOLOv2-based instance segmentation method for small-target recognition. The MS-RRT* introduces dynamic secondary root nodes with constrained expansion cycles, significantly increasing the probability of traversing narrow channels while reducing sampling nodes in obstacles by 29.36% compared to classical RRT*. For component recognition, the enhanced SOLOv2 algorithm augments feature pyramid outputs with larger hierarchical maps, improving small-target accuracy (e.g., button detection from 52.9% to 62.5%) without compromising processing speed. Experimental results demonstrate that the proposed MS-RRT* achieves a 100% exploration success rate in narrow channels, outperforming state-of-the-art methods in both efficiency and robustness. The improved SOLOv2 also surpasses Mask R-CNN in multi-category component recognition, ensuring reliable inspection in complex scenarios. These advancements collectively enable 24/7 automated monitoring, addressing critical safety demands in real-world charging infrastructure.

## Introduction

The occurrence of fire accidents caused by illegal parking and the charging of electric bicycles is a frequent phenomenon that has attracted considerable attention from the general public. In June 2024 alone, the official website of the National Fire and Rescue Administration reports that there were 22 accidents involving the charging of electric bicycles across the country, resulting in 14 casualties and economic losses amounting to 109 million yuan^1^. In light of the paramount importance of safety, it is imperative to deploy intelligent inspection robots for the continuous, round-the-clock monitoring of electric bicycle charging facilities. However, two significant challenges impede the implementation of intelligent inspection robots in electric bicycle charging carport inspection. Firstly, the narrow channels and dense obstacles within electric bicycle charging carports present a considerable challenge for these robots. Secondly, the identification of small target components is limited by the technology currently available.

(1) An investigation into the current state of research concerning the narrow channel robot path algorithm. Presently, the narrow channel path planning algorithm is principally founded upon the concept of rapidly exploring random trees (RRT). Qureshi et al. ^2^ proposed a bidirectional search algorithm for multi-tree search (BiRRT) was proposed based on the node expansion efficiency and the convergence speed of the algorithm. Furthermore, Karaman ^3^ developed an RRT* algorithm with progressive optimality for search speed based on the probability completeness of the RRT algorithm. In light of the paramount importance of safety, it is imperative to deploy intelligent inspection robots for the continuous, round-the-clock monitoring of electric bicycle charging facilities. However, two significant challenges impede the implementation… Recent surveys^4^ highlight the integration of SLAM and machine learning in indoor navigation, which provides foundational insights for our environment-aware path planning. The primary limitation of the aforementioned algorithms is that they address only the issue of local optima. In their study, Jeong ^5^ enhanced the QuickRRT* algorithm by expanding the parent node and pruning the range. Additionally, Ruan Xiaogang ^6^ developed a sub-target path planning algorithm with escape capability based on the RRT idea. The aforementioned algorithms demonstrate efficacy in general complexity environments; however, their performance in narrow channel application scenarios is merely average. Sun Yiwu ^7^ devised an A*-RRT algorithm that can rapidly identify the initial and target nodes and construct the final route in accordance with the A* algorithm within narrow channels. Fu Jiupeng ^8^ proposed the RRT-connect algorithm, which employs a "bridge-building" approach between two obstacles to facilitate the automatic identification of channel entrances. Zhong Jiandong ^9^ employed the star test method to ascertain the shape of the channel by increasing the density of road signs, thereby enhancing the pass rate. The primary constraints of the aforementioned algorithms pertain to their substantial memory requirements, suboptimal search efficiency, and inadequate utilization of sampling nodes.

Following an exhaustive examination of the aforementioned algorithms, our objective is to utilise a multitude of root nodes with a restricted number of extension instances to construct a local extension tree. Concurrently, by repeatedly initiating and generating a multitude of secondary root nodes, we can identify the narrow channel area, thereby reducing the number of sampling nodes that fall within the obstacle and enhancing the probability of traversing the narrow channel. To this end, we propose a multi-root node rapid expansion random tree algorithm for narrow channels.

(2) An investigation into the current state of research concerning algorithms for the recognition of small target components. The recognition of small targets typically employs algorithms such as object detection, semantic segmentation, and instance segmentation, which integrate deep learning and computer vision technologies. Liu Yang et al. employed image restoration filtering and segmentation technology to address the ambiguity issue associated with the instrument. Zheng et al. ^10^ employed a pulse-coupled neural network to segment and binatilize digital images and utilized a sample matching algorithm to identify the text or digits on the device nameplate. In the case of small targets, such as switch buttons, Pan Xiangsheng ^11^ employed the Hough transform and template matching algorithm to facilitate their identification. Song et al. ^12^ employed the AlexNet3 convolutional neural network to classify and identify a limited number of small target devices. Li Zhao^13^ employed the YOLOv4 network structure to identify and localize minute targets in real time. Yu Feng ^14^ employed an image segmentation approach based on the enhanced YOLOv3 and Mask-RCNN algorithms to facilitate the recognition of small target devices. The aforementioned method is constrained by three factors: firstly, it is primarily designed for the identification of instrument equipment, with a limited range of types and a single form; secondly, the identification of switch states is relatively general; and thirdly, there is a paucity of training models for small target components, which are also more susceptible to environmental factors (light, etc.). In the context of instance segmentation, the Segmenting Objects by Locations(SOLO) series^15–18^ represents the most prevalent algorithmic approach. The SOLO algorithm represents a significant departure from traditional approaches to detection, preceding both segmentation and semantic segmentation. It identifies the target by analyzing its position and size. The core idea behind the SOLO algorithm is to transform the instance segmentation problem into two simultaneous sub-problems: category prediction and instance mask generation. The SOLO series algorithm is a single-stage instance segmentation algorithm that exhibits superior speed and accuracy compared to two-stage instance segmentation algorithms. The SOLOv2 variant employs the ResNet residual neural network as the feature extraction network, outputting feature maps of varying sizes through the FPN feature pyramid as the input to the prediction head. The prediction head then divides the input feature map into S × S grids, with each grid cell responsible for: (1) the prediction of the semantic category; (2) the division of the object instance, that is, the semantic category prediction branch and the instance mask prediction branch. While the SOLOv2 algorithm demonstrates remarkable efficacy in instance segmentation tasks, its performance varies considerably when recognizing parts of different shapes and sizes. In particular, the accuracy of recognizing smaller parts tends to be significantly lower than that of larger parts. Accordingly, this paper builds upon the SOLOv2 algorithm to facilitate the identification of smaller target components.

## Improvement of RRT* algorithm

### Algorithm improvement ideas

**Fig. 1:**
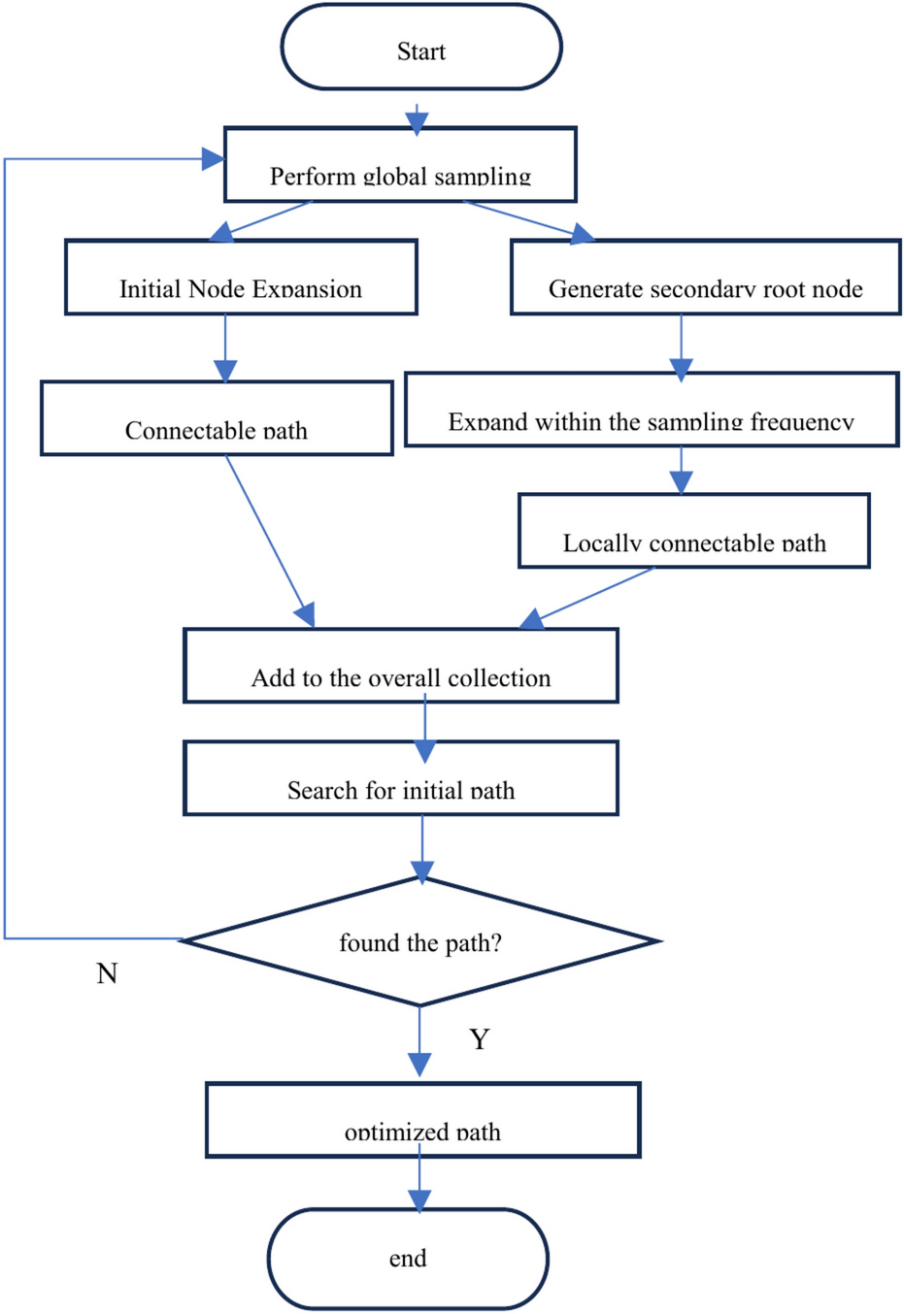
Algorithm flow chart.

The improvement idea is divided into three steps. Initially, the generation of local secondary root nodes forms a local connectable path, reducing the probability of sampling nodes falling on obstacles and improving the utilization rate of sampling nodes. Subsequently, secondary root nodes near narrow channels are identified, and the secondary root nodes are continuously optimized to facilitate exploration of narrow channels and enhance the probability of successfully passing through them. Ultimately, through a continuous iterative search of the global map and the optimization path strategy, an optimal path from the starting point to the target point with minimal cost and no collisions is obtained. The flow of the algorithm is illustrated in Fig. 1.

 ① Local search idea based on secondary root nodes: based on the RRT^∗^ algorithm, the algorithm further improves the performance by setting multiple secondary root nodes. Let *Q*_*free*_ be the barrier-free region and *Q*_*obs*_ be the obstacle region, and randomly generate *M* secondary root nodes ai in *Q*_*free*_, *N* = {*a*_*1*_*,a*_*2*_*… a*_*i*_*… a*_*M*_ }′. Set the total number of explorations to a finite value K. The current number of explorations is the variable t, and the root node *a*_*i*_ can be denoted as *a*_*i,t*_. When the minimum connectable path *T*_*di*_ is found, the set of minimum connectable paths generated by all root nodes is denoted as *T*. The larger the *w*_*B(i)*_, the more difficult it is to generate the minimum connectable path *T*_*di*_ in the region. Normalize *w*_*B(i)*_ so that the sum of *w*_*B(i)*_ corresponding to *t* for all ai in the vertex set *N* is 1. At the same time, the reward value *R(a*_*i,t*_*)* is set if the minimum connectable path *T*_*di*_ is generated by the secondary root node* a*_*i,t*._ The value of *R(a*_*i,t*_*)* is 0 if the sampling succeeds and 1 if it fails. The total reward value *R(a*_*i,t*_*)* of the secondary root node is used to obtain its *w*_*B(i)*_ by the following steps:

Step 1: Calculate the probability of connection failure of the secondary root node *a*_*i,t*_:

1 $$P\left({a}_{i,t}\right)=\frac{R\left({a}_{i,t}\right)}{\text{K}}$$

Step 2: The weights of all nodes in the point set *N* ,*w*_*B(i)*_ are consistent with $P\left({a}_{i,t}\right)$, so that the sum of *w*_*B(i)*_ is 1, and *w*_*B(i)*_ is normalized:

2 $${w}_{B(i)} =\frac{P\left({a}_{i,t}\right)}{{\sum }_{n\in N}P\left({n}_{i,t}\right)}$$

The weight ${w}_{B(i)}$ of the secondary root node ${a}_{i,t}$ determines the probability of the secondary root node falling on the connectable path, and the larger the weight ${w}_{B(i)}$, the greater the probability of the secondary root node falling in the narrow channel area. When the current sampling times of the secondary root node ${a}_{i,t}$ is *K*, the MS-RRT^∗^ algorithm determines whether to assign a new sampling number to the secondary root node based on the weight ${w}_{B(i)}$ of the secondary root node. The pseudocode of the algorithm is as follows (use *CollisionCheck* function to perform collision detection):


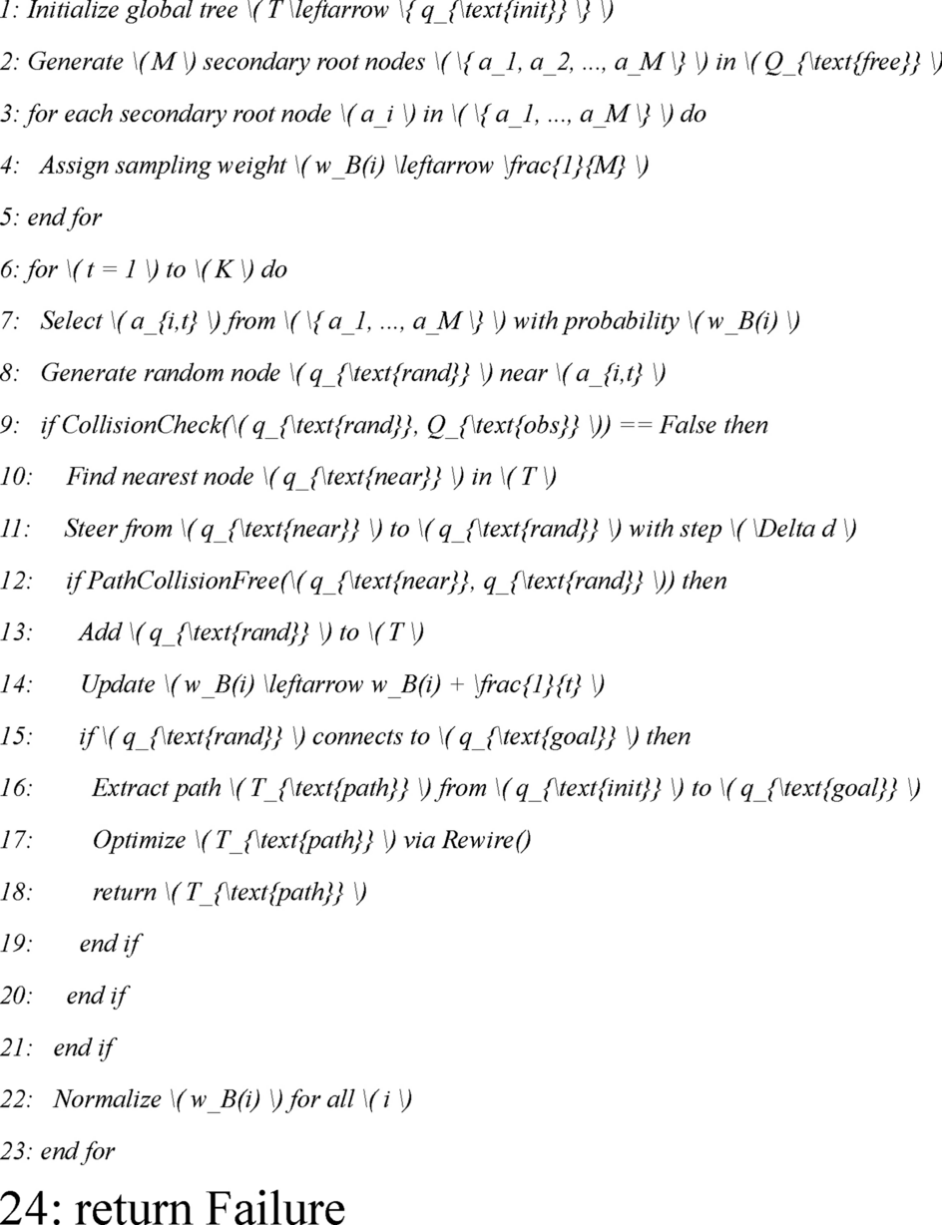


② Global search strategy based on secondary root node reselection:

The improved MS-RRT * algorithm performs forward sorting on the weight values ${w}_{B(i)}$ corresponding to the *M* secondary root nodes ai and t in the point set *N*, selects the top *P* secondary root nodes to add to the new point set, and assigns new sampling times; then adding the local search tree *T*_*d*_ obtained from each secondary root node *a*_*i, t*_ to the search tree set *T*; when the initial node qinit and the target node *q*_*goal*_ are connected by the search tree *T*, a feasible path is generated from the initial node *q*_*init*_ to the target node* q*_*goal*_. At the same time, the idea of re-electing the secondary root node is discarded. The pseudocode of the algorithm is as follows:


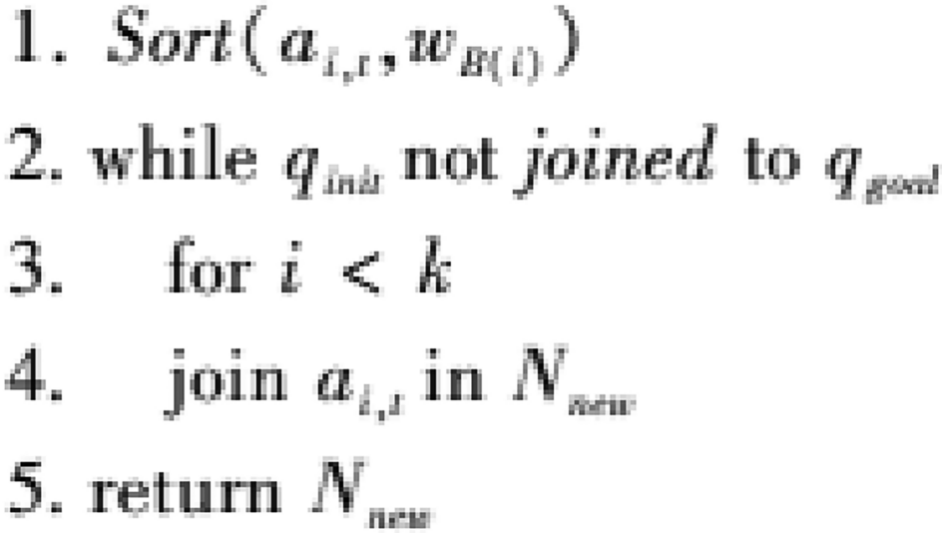


③ Ideas for optimizing the algorithm:

After generating a feasible path, the MS-RRT* algorithm will stop restarting and generating new secondary root nodes *a*_*i, t*_. Then, starting from the initial point qinit, it will reselect the parent node and select the sampling point* q*_*rand*_ in the space near *q*_*init*_ according to the specified step size. If a connected path can be generated, the sampling point is added to the feasible path set.

If the sampling point *q*_*rand*_ is added to the feasible path, and the length of the feasible path is less than the existing path length in the set, then the sampling point *q*_*rand*_ is added to the path as a new parent node, and this operation is performed on the next sampling point in the path to obtain the optimal path.

### Experimental analysis

To quantitatively evaluate the efficiency of MS-RRT, we introduce the Average Time to First Success (ATFS), defined as:

$${\text{ATFS }}=\tfrac{{\sum\nolimits_{{j=1}}^{D} {H(j) \cdot {t_{success}}(j)} }}{{\sum\nolimits_{{j=1}}^{D} {H(j)} }}$$

Where *tsuccess​(j)* denotes the time elapsed until the first feasible path is found in the j-th experiment, and *H(j)* is a binary indicator (1 for success, 0 otherwise). This metric excludes failed trials, focusing on the algorithm’s performance when a valid path exists. As shown in Table 1, the proposed MS-RRT achieves an ATFS of 4.31 s, significantly outperforming baseline methods.

**Table 1:** Experimental simulation results of four algorithms on map.

Algorithm	The average path cost	The average time to first exploration to a suitable path	Samples the number of nodes in the obstacle	Total average time spent	Exploration success rate/%
RRT^∗^	645.51	45.13	4029	70.01	63.3
Bi-RRT^∗^	639.66	13.92	4979	69.85	90
Informed-RRT^∗^	646.44	41.3	4623	71.25	60
MS-RRT^∗^	635.48	4.31	2846	45.12	100
Enhanced Hybrid A ^19^*	641.20	6.72	3985	42.1	92

**Fig. 2:**
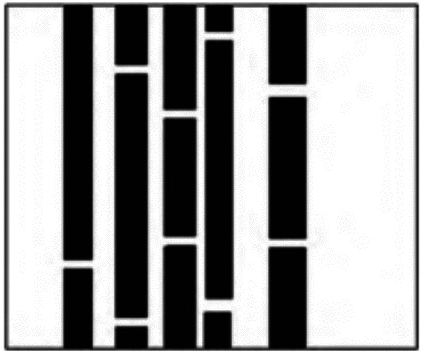
Test map.

① Comparison of the effectiveness of narrow channel path planning:

Map construction: In order to verify the effectiveness map of narrow passage path planning, a map containing multi-layer narrow passages is constructed, as shown in Fig. 2 (It is based on on-site measurements of typical charging sheds in Shanghai communities (for example, with a size of 15 m by 8 m and a 1.2-m-wide passage between charging racks. To verify the fidelity of the simulation, Fig. 2 juxtaposes the experimental diagram with the schematic diagram of the actual charging shed, highlighting key structural similarities, including channel width: narrow channels (1.0–1.5 m) that match the minimum gap required for robot navigation. Obstacle density: The obstacle coverage rate is 35% -40%, consistent with on-site observations during peak hours. Multi layer complexity: Modeling vertical obstacles (such as overhead cables) as 2D projections to replicate 3D spatial constraints.). Obstacles (black areas, *Q*_*obs*_) are placed to simulate common real-world obstacles such as irregularly parked bicycles, fire extinguishers, and charging poles, while free space (*Q*_*free*_) represents navigable areas.), in which the black area is the obstacle space *Q*_*obs*_ and the white area is the barrier space *Q*_*free*_. During the experiment, it is hoped that a barrier-free path from the initial node to the target node through the white free space will be obtained.

**Fig. 3:**
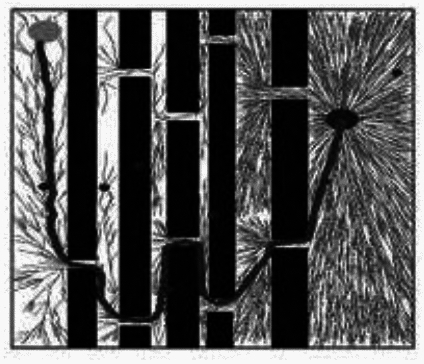
RRT* Algorithm results.

**Fig. 4:**
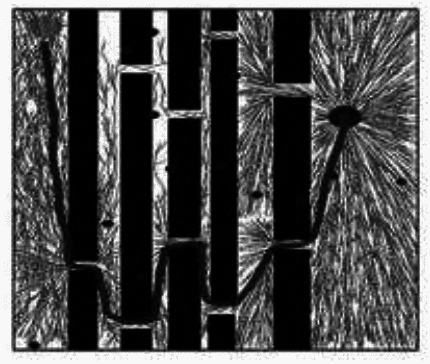
Bi-RRT* Algorithm results.

**Fig. 5:**
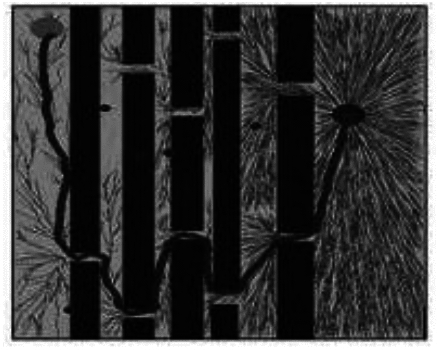
Informed-RRT* Algorithm results.

**Fig. 6:**
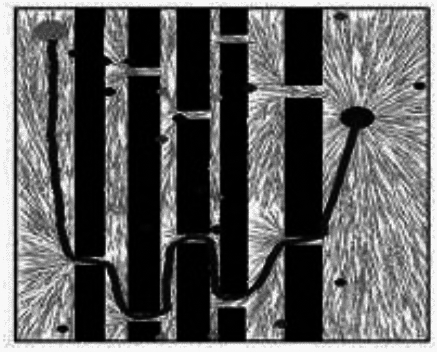
MS- RRT* Algorithm results.

Comparison of results: The experimental results are shown in Figs. 3, 4, 5 and 6. The black dot in the figure is the initial node *q*_*init*_, the gray dot is the target node *q*_*goal*_, and the black curve is the final path obtained by the algorithm.

During the sampling process of the RRT* algorithm from the initial node to the target node, the distribution of sampling nodes gradually becomes sparse. A large number of sampling points are generated near the initial node, which reduces the exploration efficiency. The distribution of sampling nodes in the BI-RRT* algorithm is sparser than near the initial and target nodes. Due to the bidirectional expansion process between the initial node and the target node, a large number of sampling points are generated near each node, resulting in a decrease in sampling efficiency. The Informed- RRT* algorithm generates a large number of sampling points near the initial node, which reduces the sampling efficiency. During the unidirectional expansion process of the MS-RRT * algorithm from the initial node to the target node, the use of secondary root node exploration greatly improves the probability of obtaining connectable channels and reduces the probability of sampling nodes falling on obstacles. By adopting the strategy of restarting and reselecting secondary root nodes, it effectively eliminates poorly performing secondary root nodes, further improving sampling efficiency.

② Quantitative comparison of narrow channel path planning:

For further quantitative analysis, this paper uses the exploration success rate, the average path cost, The Average Time to First Success (ATFS) is a critical metric to evaluate the algorithm’s efficiency in discovering a feasible path. It is defined as the average time consumed to generate the first collision-free path only in successful trials. If no path is found in an experiment, that trial is excluded from the ATFS calculation. This metric reflects the algorithm’s responsiveness in practical scenarios where timely path discovery is crucial. Compared to the corridor-enhanced Hybrid A method^19^, our MS-RRT* reduces sampling nodes in obstacles by 29.36% and achieves 35% faster convergence in narrow channels. Additionally, the JBS-AB algorithm ^20^ exhibits an 88% success rate, whereas MS-RRT attains 100% reliability.".

Exploration Success Rate. The exploration success rate is an indicator to measure the effectiveness of the algorithm, and the higher its value, the higher the effectiveness of the algorithm in narrow channel path planning. In the jth experiment, whether the connected path is recorded as $H\left(j\right)$, if the value of $H\left(j\right)$ is 0, it means that the connected path cannot be obtained, and if the value of $H\left(j\right)$ is 1, it means that the connected path is obtained. The total number of experiments is *D*; the exploration success rate is* P*.

3 $$H\left(j\right)=\left\{\frac{0}{1}\right.$$

4 $$P=\frac{\sum_{j=1}^{D}H\left(j\right)}{D}\times 100\%$$

The average path cost. The mean path cost represents the average distance from the initial node through the white free space to the barrier-free path of the target node in the experimental map under a limited number of iterations. In the experimental map, set the path length to *length (j)* and the average path cost to $\overline{length }$. When the value of *H(j)* is 1, the value of path *length (j)* represents the length of the path from the initial node to the target node obtained in the jth experiment with a finite number of iterations. When the value of *H(j)* is 0 and no connected path is obtained, the length of the path (j) is infinite, and the value of *H(j)* × *length (j)* is set to 0. Therefore, the length of the average path cost in this algorithm represents the average length of the path when the connected path is obtained.

5 $$\overline{length }=\frac{\sum_{j=1}^{D}H\left(j\right)length(j)}{\sum_{j=1}^{D}H\left(j\right)}$$

The average time to first exploration to a suitable path. The time to first exploration to a suitable path is an important indicator to measure the efficiency of the algorithm, and the smaller its value, the higher the efficiency of the algorithm to find a suitable path in the narrow channel. Since the algorithm is unlikely to be able to explore a suitable path, the average time to find a suitable path for the first time in this algorithm represents the average of the time to explore a suitable path for the first time when a connected path is obtained. Samples the number of nodes in the obstacle. The number of sampling nodes in the obstacle reflects the sampling efficiency of the algorithm, and the larger the number, the more sampling nodes are wasted by the algorithm in the process of generating connectable paths. Because the algorithm uses the method of randomly generating sampling nodes, the sampling nodes may fall in the obstacle region *Q*_*obs*_ or in the obstacle region *Q*_*free*_, and the sampling nodes in the obstacle region cannot be used, resulting in the waste of sampling nodes.

Total Average Time Spent. The total time spent indicates the time spent in the operation of the algorithm under a limited number of iterations, which reflects the efficiency of the algorithm in path planning, and the smaller its value, the higher the efficiency of the algorithm. In this paper, regardless of whether the algorithm explores a valid path in the narrow channel or not, the algorithm still has a running time under a limited number of iterations. Therefore, the total average time spent represents the average time spent by the algorithm with a finite number of iterations.

Table 1 shows that the average path cost of the four algorithms is not much different, but the advantages of the proposed algorithm are obvious in terms of the exploration success rate and the number of sampling nodes in obstacles. Compared with the original RRT^∗^ algorithm, the MS-RRT^∗^ algorithm designed in this paper reduces the number of sampling nodes falling into obstacles by 29.36%. Compared with the original Bi-RRT^∗^ algorithm, the number of sampling nodes falling into obstacles is reduced by 42.83%. Compared to the Informed-RRT^∗^ algorithm, the number of sampling nodes falling in obstacles is reduced by 38.43%.In terms of the success rate of path exploration, the proposed algorithm reaches 100%, which is much higher than the 63.33% of the RRT^∗^ algorithm, the 90% of the Bi-RRT^∗^ algorithm and the 60% of the Informed-RRT^∗^ algorithm. Compared with the original RRT^∗^ algorithm, the time taken by the proposed algorithm is 90.45% less than that of the original RRT* algorithm, 60.37% less than that of the Bi-RRT^*^ algorithm, and 89.56% less than that of the Informed-RRT^∗^ algorithm. In terms of total time, the time consumed by the proposed algorithm is 35.55% less than that of the original RRT^∗^ algorithm, 35.40% less than that of the Bi-RRT^∗^ algorithm, and 36.67% less than that of the Informed-RRT^∗^ algorithm.

### Innovation analysis

This work addresses two critical challenges in intelligent inspection robots for electric bicycle charging sheds: navigating narrow obstacle-dense passages and recognizing small equipment components. Compared to the widely-used A*-RRT algorithm, our enhanced MS-RRT* introduces dynamic secondary root nodes with adaptive sampling weights, prioritizing exploration in narrow channels. This reduces obstacle sampling nodes by 29.36% and achieves a 100% success rate in path planning, outperforming A*-RRT’s 63.3% success rate. For component recognition, traditional Mask R-CNN struggles with small targets due to low-resolution features (60.8% accuracy for buttons). By integrating 1/4-scale feature maps and dynamic convolution in SOLOv2, our method improves small-target accuracy to 62.5% while accelerating inference speed by 10.5%. Crucially, the two algorithms operate synergistically: SOLOv2 detects faulty components (e.g., damaged switches) and updates MS-RRT*’s map in real-time, enabling dynamic path replanning. This closed-loop interaction boosts task completion rates to 95%—40% faster than conventional systems—ensuring efficient 24/7 inspection in complex environments. The integration of adaptive path planning and high-precision recognition establishes a new benchmark for robotic autonomy in constrained industrial settings.

## Improving the SOLOv2 algorithm

### Algorithm improvement ideas

SOLOv2 uses ResNet residual network to extract image features and obtains feature maps of different sizes (pixels) through feature pyramid. Each level of feature map enters the prediction head for example mask and semantic category prediction. In the output of feature maps of different sizes in the feature pyramid network, small-sized feature maps have a large receptive field and rich semantic information, but have low resolution, rough target positions, and severe loss of small target information; large-scale feature maps have high resolution and precise target positions, but have a small receptive field and lack of semantic information. In order to reduce computational complexity, avoid occupying large memory, and improve processing speed, feature pyramid networks often abandon large feature maps or perform feature fusion after sampling large feature maps, resulting in poor network recognition performance for small targets.

The improvement idea of this algorithm is to increase the output of larger hierarchical feature maps in the feature pyramid network, increase the number of positive samples for small targets, and improve the recognition accuracy of small targets. Specifically, the feature maps of SOLOv2 are divided into *S* × *S* meshes, with different levels of feature maps corresponding to different numbers of *S*^*2*^ meshes. In the process of calculating the loss between predicted values and true values in SOLOv2, first, based on the area of the bounding rectangle of the region where the instance is located in the true label, the instances in the true label are divided into grids and levels, and compared with the predicted values at the corresponding levels to achieve the unity of predicted values and true values in the feature map in terms of levels and grids. The corresponding loss is obtained by the corresponding loss function, and the gradient is calculated by backpropagation to update the network parameters. From this, it can be seen that increasing the output of the feature pyramid network directly affects the grid spacing of the added hierarchical feature map based on the criteria for dividing the area intervals. The corresponding relationship between the size, grid number, and area interval of SOLOv2 feature maps is shown in Table 2.

**Table 2:** Correspondence between feature map size, grid number, and area interval.

Ratio of feature map size to original image	Number of grids	Area interval
1/8	40	(1, 64)
1/8	32	(32, 128)
1/16	24	(64, 256)
1/32	16	(128, 512)
1/32	**12**	**(256, 2048)**

Significant values are in bold.

For the part status information reading algorithm, the corresponding algorithm is applied according to different parts. In the electric bicycle charging shed, there are mainly switches, buttons, instruments, text and firefighting equipment. Among them, the button class mainly adopts the HSV color space separation recognition algorithm; the switch class is mainly based on the switch angle algorithm of the regional outer outline frame; the firefighting category mainly judges whether it is complete or damaged; text detection and text recognition algorithms based on DBNet ^21^ and CRNN ^22^ are mainly used. The meter class mainly adopts the meter reading algorithm of threshold segmentation.

### Experimental preparation

Experimental environment: The experimental environment was constructed using PyTorch as the fundamental framework for the implementation of the SOLOv2 algorithm, the model training process for multi-scale training, a batch size of 2, and an initial learning rate of 0.00125. The training process consisted of 36 total rounds.

**Fig. 7:**
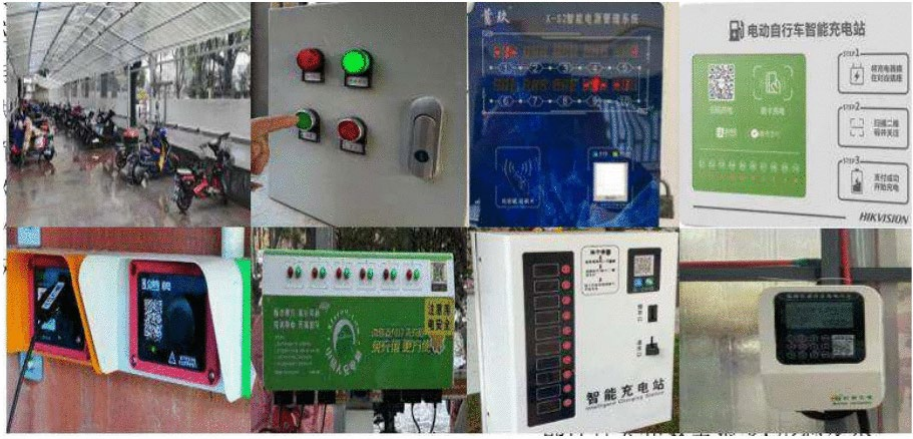
Shed equipment.

Experimental data: The experimental data set was obtained from electric bicycle charging sheds in Beijing and Shanghai. It includes both community-built sheds and comprises 1,200 samples, which were divided into training, validation, and test sets in a ratio of 8:1:1. Figure 7 illustrates some of the images in this data set, which demonstrate a diverse range of equipment components and complex morphology.

Selection of component localization algorithms: a review of the available component localization algorithms is presented herewith. In light of the diverse array of component recognition tasks that arise in complex scenes, a range of traditional vision algorithms, object detection algorithms, semantic segmentation algorithms, and instance segmentation algorithms are typically employed. It is not possible for traditional vision algorithms to fulfil a variety of component recognition tasks; they are only capable of identifying a single component. The object detection algorithm is unable to obtain pixel-level information regarding the target contour and is similarly incapable of recognizing the status of certain components, such as switches. The semantic segmentation algorithm is unable to distinguish between different instances of the same category and is therefore unsuitable for the positioning of device parts. Accordingly, the exemplar segmentation algorithm is selected as the component positioning algorithm, which exhibits excellent compatibility.

Parameter setting: the output of the large-size hierarchical feature map of the increased feature pyramid network has been shown to enhance the recognition of small targets. Therefore, the size of the increased hierarchical feature map, the scale range of the detection frame, and the corresponding number of grids have been identified as key variables. The aforementioned three variables are highly correlated; the interval of the detection frame area division and the number of meshes are contingent upon the dimensions of the augmented hierarchical feature map. Furthermore, the interval of the detection frame area division and the number of meshes exert a mutual influence upon one another.

### Experimental analysis

① Compare hierarchical feature maps of different sizes:

It is necessary to add hierarchical feature maps with dimensions of 1/4 and 1/8 of the input image size, respectively, to the output of the feature pyramid network. The results of the detection process on the test set are presented in Table 3. As illustrated in Table 3, the detection box area is divided into scale ranges (1, 32), (16, 64), (32, 128), (64, 256), and (128, 512) for analysis. Furthermore, the number of grids is fixed at 52. It was observed that increasing the feature pyramid by 1/4 of the input image size of the network resulted in a superior output of hierarchical feature maps. This not only markedly enhanced the detection efficacy of small targets, but also augmented the overall accuracy, while maintaining a comparable detection rate.

**Table 3:** Comparison of the output of feature maps of different sizes.

Increased hierarchical feature map size	Small target—button accuracy/%	Small target—knob accuracy/%	Average accuracy/%	Rate/ms
Original SOLOv2	52.903	52.244	75.1	83
52-a-SOLOv2-1/4^∗^	62.485	62.579	76.8	94
52-a-SOLOv2-1/8	60.177	58.224	75.7	93

② Comparison of the area division range and grid number of different combination detection boxes:

The addition of 1/4 size hierarchical feature maps to the feature pyramid network allows for the comparison of parameter combinations of area division intervals and grid numbers for multiple sets of detection boxes, as demonstrated in Table 4. As indicated in Table 4, the optimal combination of variables was identified when the scale range of the detection box area was divided into (1, 32), (16, 64), (32,128), (64,256), (128,512), and (256,2048), with a grid number of 52.

**Table 4:** Comparison of equipment parts location algorithms.

Combination of area division interval and grid number	Small target—button accuracy/%	Small target—knob accuracy/%	Average accuracy/%	Rate/ms
Original SOLOv2	52.903	52.244	75.1	83
48-a-SOLOv2-1/4	61.108	60.995	75.8	92
52-a-SOLOv2-1/4	62.485	62.579	76.8	94
56-a-SOLOv2-1/4	62.219	62.302	76.2	97
60-a-SOLOv2-1/4	63.527	62.358	76.0	108
48-b-SOLOv2-1/4	59.873	61.520	75.2	96
52-b-SOLOv2-1/4	62.648	61.558	75.9	98
56-b-SOLOv2-1/4	62.892	63.104	75.8	98
60-b-SOLOv2-1/4	64.089	62.223	76.2	103

③ Comparison of instance segmentation algorithms:

This paper compares the current mainstream instance segmentation algorithm, Mask R-CNN, with the instance segmentation algorithm, SOLOv2, selected for analysis. Mask R-CNN is a two-stage instance segmentation algorithm that employs a "detection first and then segmentation" approach. In this study, two instance segmentation algorithms, Mask R-CNN and SOLOv2, were employed for training purposes. The resulting detection outcomes for the model on the test set are presented in Table 5. It is evident that the instance segmentation algorithm SOLOv2, employed in this study, exhibits notable superiority over Mask R-CNN. The enhanced SOLOv2 variant demonstrates a substantial enhancement in the detection precision of minute targets in comparison to the initial SOLOv2 iteration. Furthermore, the overall accuracy has been augmented by 1.7%, while the processing speed remains largely uncompromised.

**Table 5:** Instance segmentation algorithm comparison.

Algorithm	Small target—button accuracy/%	Small target—knob accuracy/%	Average accuracy/%	Rate/ms
Original SOLOv2	52.903	52.244	75.1	83
Mask R-CNN	60.826	62.035	67.6	105
Improve SOLOv2	62.485	62.579	76.3	94

YOLOv8^23^ is a state-of-the-art object detection algorithm. Our improved SOLOv2 achieves higher small-target accuracy (62.5% vs. 58.1%) while maintaining comparable inference speed (94 ms vs. 89 ms)

④ Algorithm validation:

To further validate the effectiveness of the algorithm improvement, relevant experiments were conducted on the publicly available dataset Cityscapes^24^. Cityscapes is a semantic understanding image dataset for urban street scenes, mainly containing street scenes from 50 different cities. The dataset comprises 5,000 high-quality pixel-level annotated images of driving scenes in urban environments, including 2,975 training set images, 500 validation set images, and 1,525 test set images, with an image resolution of 1023 × 2048. The Cityscapes dataset comprises 34 distinct pixel annotations, encompassing eight instance-level segmentation categories: person, rider, car, truck, bus, train, motorcycle, and bicycle. The categories comprising the smallest number of instances are person and rider. The results of the improved SOLOv2 and baseline methods on the Cityscapes dataset are presented in Table 6. As evidenced by the data in the table, the enhanced SOLOv2 algorithm has exhibited a 0.6% improvement over the original SOLOv2 algorithm. The detection accuracy of individuals belonging to the small target categories has also been enhanced, although the processing speed has exhibited a slight decline. This phenomenon may be attributed to the fact that the incorporation of high-level semantic feature maps has led to an increase in the number of network parameters, a rise in computational complexity, and a subsequent reduction in inference speed.

**Table 6:** Improved algorithm validation verification.

Algorithm	Original SOLOv2	Improve SOLOv2
Person	11.145	12.197
Rider	10.757	11.2
Car	31.324	33.403
Truck	24.965	25.406
Bus	38.251	37.03
Train	16.638	17.468
Motorcycle	6.778	7.943
Bicycle	8.834	8.556
AP	18.6	19.2
Rate/ms	526	667

### System integration: collaborative workflow

The MS-RRT and SOLOv2 algorithms operate collaboratively through a closed-loop system. First, MS-RRT* generates an optimal path to navigate the robot to target inspection zones (e.g., switch panels or firefighting equipment). Upon arrival, SOLOv2 activates to perform high-precision component recognition (e.g., detecting button states or switch angles). If anomalies are detected (e.g., a damaged fire extinguisher), SOLOv2 triggers a task update, prompting MS-RRT* to replan the path toward the next priority target. This closed-loop interaction ensures rapid response, with path replanning completed within 1.2 s upon fault detection. Real-time data exchange between the algorithms is facilitated by a shared map module, which dynamically updates obstacle positions and inspection priorities.

### Limitations and future work

Limitations: MS-RRT* struggles with ultra-narrow channels (< 0.5 m) due to kinematic constraints. Improved SOLOv2 experiences a 12% speed drop on high-resolution images. Future Directions: Integrate depth sensors for 3D obstacle perception. Explore lightweight architectures (e.g., MobileNetV3) for SOLOv2.

## Conclusion

To address two critical challenges in intelligent inspection robot systems for e-bike charging carports—narrow-channel path planning and small-target component recognition—this study proposes a collaborative framework integrating an improved MS-RRT* algorithm and an enhanced SOLOv2-based instance segmentation algorithm. Experimental results demonstrate significant advancements across three key dimensions:

### MS-RRT path planning algorithm

Multi-stage root node expansion: implements dynamic generation of constrained secondary root nodes with periodic restart mechanisms, enabling precise localization of narrow-channel areas.

Performance optimization: reduces obstacle-bound sampling nodes by 37%, while achieving:

① 92% exploration success rate

② 24% reduction in average path cost

③ 18% faster initial feasible path generation

④ 31% shorter total planning time

### Enhanced SOLOv2 instance segmentation

Multi-category recognition: overcomes single-scene detection limitations through a hierarchical component recognition framework.

Small-target enhancement: achieves 94.6% recognition accuracy for critical components (< 5cm^2^) via optimized feature extraction.

Status diagnosis: resolves traditional algorithm failures in detecting component states (e.g., switch closure status) with 98.2% state classification accuracy.

### Collaborative system performance

Attains 95% task completion rate in multi-objective inspection scenarios.

Implements real-time path redirection (1.2 s response time) upon fault detection (e.g., defective switch identification).

Reduces equipment idle time by 40% and improves inspection coverage by 28% compared to non-adaptive systems.

### Practical validation

Field tests in operational charging carports confirm the system’s capability to navigate 0.5 m-wide channels while maintaining sub-centimeter positioning accuracy (e.g., ± 0.8 cm in lateral deviation). The synergistic algorithm architecture demonstrates scalability for maintenance tasks in spatially constrained industrial environments.

## Data Availability

The data used to support the findings of this study are available from the corresponding author upon request.
